# Influenza versus other respiratory viruses – assessing severity among hospitalised children, Belgium, 2011 to 2020

**DOI:** 10.2807/1560-7917.ES.2023.28.29.2300056

**Published:** 2023-07-20

**Authors:** Natalie Fischer, Sarah Moreels, Nicolas Dauby, Marijke Reynders, Evelyn Petit, Michèle Gérard, Patrick Lacor, Siel Daelemans, Bénédicte Lissoir, Xavier Holemans, Koen Magerman, Door Jouck, Marc Bourgeois, Bénédicte Delaere, Sophie Quoilin, Steven Van Gucht, Isabelle Thomas, Nathalie Bossuyt, Cyril Barbezange

**Affiliations:** 1 National Influenza Centre, Sciensano, Brussels, Belgium; 2 European Public Health Microbiology Training Programme (EUPHEM), European Centre for Disease Prevention and Control (ECDC), Stockholm, Sweden; 3 Health Services Research – Epidemiology and Public Health, Sciensano, Brussels, Belgium; 4 Centre for Environmental Health and Occupational Health, School of Public Health, Université Libre de Bruxelles (ULB), Brussels, Belgium; 5 Centre Hospitalier Universitaire St-Pierre, Brussels, Belgium; 6 Department of Laboratory Medicine, Medical Microbiology, Algemeen Ziekenhuis Sint-Jan, Brugge-Oostende AV, Belgium; 7 Internal Medicine-Infectious Diseases, Universitair Ziekenhuis Brussel, Brussels, Belgium; 8 Paediatric Pulmonary and Infectious Diseases, Universitair Ziekenhuis Brussel, Brussels, Belgium; 9 Microbiology, Grand Hôpital de Charleroi, Charleroi, Belgium; 10 General Internal Medicine and Infectiology, Grand Hôpital de Charleroi, Charleroi, Belgium; 11 Clinical Laboratory, Jessa Ziekenhuis, Hasselt, Belgium; 12 Infection Control, Jessa Ziekenhuis, Hasselt, Belgium; 13 Centre Hospitalier Universitaire UCL Namur, Yvoir, Belgium; 14 Epidemiology of Infectious Diseases – Epidemiology and Public Health, Sciensano, Brussels, Belgium

**Keywords:** influenza, non-influenza respiratory virus, burden of disease, risk factor, severity

## Abstract

**Background:**

Knowledge on the burden attributed to influenza viruses vs other respiratory viruses in children hospitalised with severe acute respiratory infections (SARI) in Belgium is limited.

**Aim:**

This observational study aimed at describing the epidemiology and assessing risk factors for severe disease.

**Methods:**

We retrospectively analysed data from routine national sentinel SARI surveillance in Belgium. Respiratory specimens collected during winter seasons 2011 to 2020 were tested by multiplex real-time quantitative PCR (RT-qPCR) for influenza and other respiratory viruses. Demographic data and risk factors were collected through questionnaires. Patients were followed-up for complications or death during hospital stay. Analysis focused on children younger than 15 years. Binomial logistic regression was used to identify risk factors for severe disease in relation to infection status.

**Results:**

During the winter seasons 2011 to 2020, 2,944 specimens met the study case definition. Complications were more common in children with underlying risk factors, especially asthma (adjusted risk ratio (aRR): 1.87; 95% confidence interval (CI): 1.46–2.30) and chronic respiratory disease (aRR: 1.88; 95% CI: 1.44–2.32), regardless of infection status and age. Children infected with non-influenza respiratory viruses had a 32% higher risk of complications (aRR: 1.32; 95% CI: 1.06–1.66) compared with children with influenza only.

**Conclusion:**

Multi-virus testing in children with SARI allows a more accurate assessment of the risk of complications and attribution of burden to respiratory viruses beyond influenza. Children with asthma and respiratory disease should be prioritised for clinical care, regardless of their virological test result and age, and targeted for prevention campaigns.

**Key public health message**

**What did you want to address in this study?**

We wanted to investigate the severity of disease in children hospitalised with severe acute respiratory infections in Belgium. More specifically, we wanted to compare the risk of developing a complication - for example pneumonia or transfer to intensive care - in children with influenza vs other common respiratory viruses, taking into account the child’s age and underlying risk factors, such as asthma, chronic lung or heart disease, obesity or diabetes.

**What have we learnt from this study?**

We found that children infected with a common respiratory virus other than influenza were more at risk of developing a complication than those with influenza. Children with asthma or chronic lung disease had twice the risk of complications, independently of their age or the respiratory virus responsible for their hospitalisation.

**What are the implications of your findings for public health?**

Our study advocates for comprehensive year-round surveillance based on sentinel systems and multi-virus testing. When several viruses circulate simultaneously, this is particularly pertinent for assessing virus-specific disease incidence and severity as well as vaccine effectiveness, especially in children. Children with asthma and chronic respiratory disease should be prioritised for prevention programmes and clinical care during hospitalisation.

## Introduction

Influenza type A and B viruses cause respiratory infections in humans that follow a seasonal pattern, coinciding with the winter months in temperate regions [1]. Infections can range from mild to severe forms that might require hospitalisation and can lead to severe complications and death. Underlying medical conditions, pregnancy and age (children < 1 year and adults ≥ 65 years) were previously described as risk factors for a severe course of influenza [2]. Especially in children, differences in severity might also be associated with co-infections with other respiratory viruses [3,4].

Taking all viral and bacterial aetiologies into account, acute respiratory infections are among the top ranked causes of death in children under 5 years globally [5]. Based on data before the COVID-19 pandemic, the global annual burden of influenza during a regular season is estimated at ca 1 billion infections, and 3–5 million of these cases develop severe illness [6]. An estimated 290,000–650,000 influenza-related respiratory deaths occur annually on a global level, with the highest mortality rate recorded among adults ≥ 75 years [7].

Nevertheless, a large proportion of the influenza-associated illness burden falls on young children and infants, especially in low and lower-middle income countries. Based on a systematic review of more than 100 studies, an estimated 109.5 million influenza episodes arose among children younger than 5 years globally in 2018, resulting in 870,000 hospital admissions and up to 34,800 deaths [8].

Vaccination with available, safe influenza vaccines is the most effective way to prevent disease and mortality and is especially recommended in vulnerable populations at high risk of severe disease. In children, the World Health Organization (WHO) recommends annual vaccination between 6 months and 5 years of age [9]. Nevertheless, childhood vaccination is not routinely established in many countries. In Belgium, the Superior Health Council recommends vaccination against seasonal influenza for children over 6 months of age with a chronic condition or under long-term acetylsalicylic acid therapy, as well as for pregnant women and people living in the same household as children under 6 months of age [10]. Vaccines for other common respiratory viruses impacting children’s health are not yet available but are in advanced development, with a vaccine against respiratory syncytial virus (RSV) already marketed in the United States for people above 65 years [11-13].

The overall prevalence of influenza in Belgium is estimated at 500,000 cases of influenza-like illness each year, corresponding to about 5% of the population [14]. An estimated one in 1,000 patients develops complications that require hospitalisation, and more than 90% of influenza-related deaths occur in patients older than 65 years. However, little is currently known about the epidemiology of children hospitalised with influenza in Belgium and how the severity of disease relates to underlying risk factors or co-infection with other respiratory viruses.

We present here the analysis of surveillance data collected through the national severe acute respiratory infection (SARI) sentinel hospital surveillance network over the influenza seasons from 2011 to 2020, with the aim to address some of these knowledge gaps.

## Methods

### Study design, population and data collection

The SARI surveillance protocol is summarised in the Supplement and has been extensively described, including the demographic and clinical data that were collected via questionnaire with each sample [15]. In brief, the Belgian SARI network is composed of six sentinel hospitals. During the influenza season, generally between epidemiological weeks 50 and 18 of the following year depending on the season, all patients matching the clinical case definition are enrolled and a respiratory sample is collected for each of them. We conducted a retrospective analysis for seasons 2011/12 to 2019/20 of the surveillance data on SARI in hospitalised children younger than 15 years in Belgium. Virological analysis of respiratory samples (mainly nasopharyngeal swabs, some nasopharyngeal aspirates and broncho-alveolar lavages) was performed at the National Influenza Centre (NIC) at Sciensano. The study case definition included: acute respiratory illness with onset within the last 10 days, with measured or reported fever of ≥ 38 °C, with cough and/or dyspnoea, with hospitalisation for at least one night, and age < 15 years.

### Descriptive data analysis

The distribution of cases was described by sex (collected as a binary value) and age group (< 1, 1–4, 5–14 years), across all winter seasons (2011–2020) and per season. We estimated the proportion of risk factors, occurrence of complications, influenza virus infection by type and subtype or lineage, and co-infection with other respiratory viruses (for samples collected from season 2015/16 onwards), and compared differences in proportions between groups. For further details on data analysis and tools used, see the Supplementary material.

### Statistical modelling

We further investigated the association between infection status and occurrence of complications using binomial logistic regression. Multivariable models were fit using the glm() function in the R statistical computing package (version 4.0.3) [16] and built following the principle of parsimony using a forward selection approach. Occurrence of complications was defined as the outcome variable, while infection status – first influenza-positive and -negative, then in a more detailed model including influenza virus infection only, co-infection, infection with other respiratory virus only, or no virus detected – was defined as the exposure variable. All models were corrected for age in years, sex and risk factors as explanatory variables. Nested models were compared with the ANOVA function and evaluated by chi-squared test. A p value of < 0.05 was considered as threshold for significance.

## Results

### Characteristics of children hospitalised with severe acute respiratory infection in Belgium

**Figure 1:**
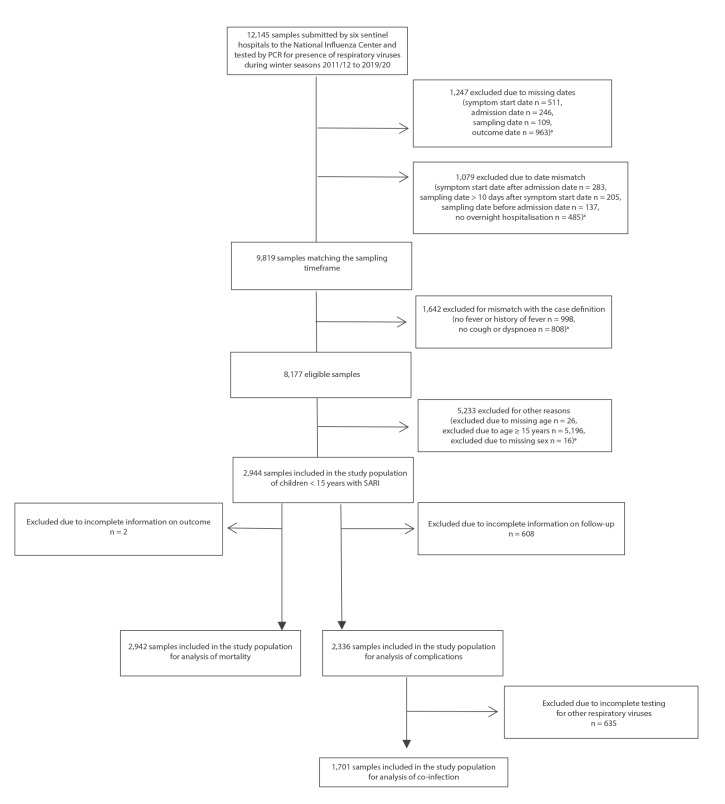
Inclusion and exclusion criteria to derive the selected study population of children < 15 years hospitalised with severe acute respiratory infection, Belgium, 2011/12–2019/20 (n = 12,145)

During the influenza seasons from 2011 to 2020, 12,145 respiratory specimens were submitted to the NIC for analysis (Figure 1). Of these, 2,944 (24.2%) came from children younger than 15 years who matched the study case definition and were retained in the analysis.

SARI: severe acute respiratory infection.

^a^ More than one reason could apply to one case.

A detailed description of study population characteristics per age group is appended in Supplementary Table S1. Among the 2,944 children hospitalised with SARI over the nine seasons, 1,327 (45.1%) were female and 1,617 (54.9%) male, and 1,279 (43.5%) were younger than 1 year, 1,252 (42.5%) between 1 and 4 years and 413 (14.0%) between 5 and 14 years. The median age was 1.2 years (interquartile range (IQR): 0.4–3.1). For 2,515 children with known information, only 111 (4.4%) were vaccinated against influenza for the ongoing season. The vaccination rate increased with age group (chi-squared test for trend p < 0.0001).

A detailed description of the risk factors and outcome status per sex and age group is appended in Supplementary Table S2. Among all children in the study population, 359 (12.2%) had underlying risk factors, among whom 10.6% (38/359) had more than one risk factor. The proportions of children with risk factors increased with age (chi-squared test for trend p < 0.0001). The most common risk factors were asthma (5.3%; 130/2,483 with known status of risk factors) and chronic respiratory disease (4.6%; 112/2,483), whose proportions also increased with age (both: chi-squared test for trend p < 0.0001).

### Influenza virus infection among children hospitalised with severe acute respiratory infection

**Figure 2:**
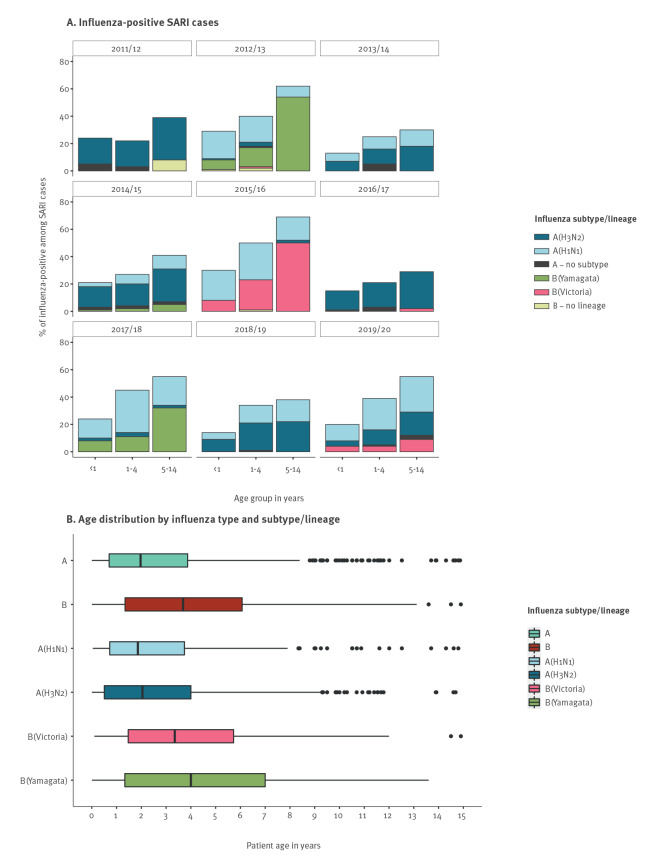
Influenza virus types and subtypes/lineages among children < 15 years hospitalised with severe acute respiratory infection, Belgium, 2011/12–2019/20 (n = 938)

Influenza positivity rates by type and subtype/lineage are listed in detail per season, sex and age group in Supplementary Table S3, per risk factor in Supplementary Table S4 and per complication in Supplementary Table S6. Across all nine seasons, 31.9% (938/2,944) of the study population tested positive for influenza, with 74.7% (701/938) positive for type A and 25.3% (237/938) for type B. Most winter seasons were dominated by type A (≥ 60% of the positive samples in seven of nine seasons), of which seasons 2011/12 and 2016/17 reached > 80% of subtype H3N2 (Figure 2A) and seasons 2012/13, 2015/16 and 2017/2018 reached > 90% of subtype H1N1 among all influenza-positive cases. Only seasons 2012/13 and 2015/16 showed co-dominance of influenza A(H1N1) virus with influenza B/Yamagata and B/Victoria, respectively (Figure 2A).

SARI: severe acute respiratory infection.

Panel A: Weeks covered by the SARI surveillance for each season: 2011/12, week 4 2012 to week 16 2012; 2012/13, week 51 2012 to week 18 2013; 2013/14, week 5 2014 to week 17 2014; 2014/15, week 1 2015 to week 17 2015; 2015/16, week 52 2015 to week 17 2016; 2016/17, week 1 2017 to week 16 2017; 2017/18, week 50 2017 to week 17 2018; 2018/19, week 1 2019 to week 15 2019; 2019/20, week 1 2020 to week 14 2020.

Panel B: Box-and-whisker plot with Tukey’s outliers as black dots.

The positivity rate for influenza was higher among females (34.5%; 458/1,327) than among males (29.7%; 480/1,617; z-test with Yates continuity correction; p < 0.01), with no difference per type, subtype or lineage. Children < 1 year had a lower influenza positivity rate (20.6%; 263/1,279) compared with children 1–4 years (37.5%; 469/1,252) and 5–14 years (49.9%; 206/413; chi-squared test for trend, p < 0.0001). This trend was observed in each winter season, regardless of the dominant strain (Figure 2A).

Overall, the median age was higher for cases infected with influenza virus type B (3.7 years; IQR: 1.3–6.1) than with type A (2.0 years; IQR: 0.7–3.9; pairwise Wilcoxon test: p < 0.0001) (Figure 2B). There was no difference in the median age of cases between the two type A subtypes or the two type B lineages.

There was no difference in the influenza positivity rate between SARI cases with (29.8%; 107/359) or without (32.6%; 693/2,124) risk factors, nor between patients with different specific risk factors.

### Severity of disease among children hospitalised with SARI

Information on the outcome was close to complete (99.9%; 2,942/2,944). The overall mortality was very low (0.2%; 7/2,942) and all deceased patients were < 5 years. The median length of hospital stay was 3 days (IQR: 2.0–5.0). Complete follow-up of complications during hospitalisation was available for 79.3% (2,336/2,944) of cases (Figure 1). The description of complications by sex, age group and risk factor is appended in Supplementary Table S5. Overall, 734 of the 2,336 patients with available information (31.4%) experienced a complication. Among those, more than three quarters developed pneumonia (77.0%; n = 565) and one quarter required respiratory assistance (25.2%; n = 185). Transfer to an intensive care unit (ICU; 7.6%; n = 56) and occurrence of acute respiratory distress syndrome (ARDS; 7.4%; n = 54) were less common, and extracorporeal membrane oxygenation was never used. Children requiring respiratory assistance (median age: 0.8 years; IQR: 0.3–1.9) were younger than children requiring ICU transfer (median age: 1.4 years; IQR: 0.4–5.2; pairwise Wilcoxon test: p = 0.01). Children developing ARDS (median age: 0.8 years; IQR: 0.2–1.1) were younger than children developing pneumonia (median age: 1.6 years; IQR: 0.8–3.6; pairwise Wilcoxon test: p < 0.0001).

Half of the patients with any risk factor experienced complications (49.7%; 149/300), more than patients without a risk factor (27.9%; 482/1,726; proportions z-test: p < 0.0001). This was especially the case for patients with asthma (56.9%; 62/109) vs patients without asthma (31.7%; 591/1,862; proportions z-test: p < 0.0001), and for patients with chronic respiratory disease (56.7%; 59/104) vs patients without chronic respiratory disease (31.8%; 595/1,870; proportions z-test: p < 0.0001).

A higher proportion of patients with any risk factor required ICU transfer (13.4%; 20/149) vs patients without risk factors (5.2%; 25/482; proportions z-test: p < 0.01). Likewise, a higher proportion of patients with any risk factor experienced ARDS (12.8%; 19/149) vs patients without risk factors (6.4%; 31/482; proportions z-test: p = 0.02).

### Association of severity with patient characteristics in influenza-positive children

Overall, the proportion of complications was lower in children who tested positive for influenza (23.4%; 180/768) than in influenza-negative children (35.3%; 554/1,568; proportions z-test: p < 0.0001). Among children experiencing complications, respiratory assistance was more often required for influenza-negative cases (27.4% (152/554) than influenza-positive cases (18.3%; 33/180; proportions z-test: p = 0.02), but pneumonia was more frequent for influenza-positive cases (88.3%; 159/180) than influenza-negative cases (73.3%; 406/554; proportions z-test: p < 0.0001).

In addition, even if the medians were identical, there was a significant difference in the distributions and influenza-negative patients were found to have a longer hospital stay than influenza-positive patients (Wilcoxon rank sum test: p < 0.001). There was no difference in length of stay between influenza virus types, subtypes or lineages.

The analysis of the risk of complications among influenza-positive children with SARI is detailed in Supplementary Table S7. Multivariable modelling showed that complications among influenza-positive children were associated with the presence of risk factors (adjusted RR (aRR): 2.85; 95% CI: 2.17–3.64; p < 0.0001) and with a younger age (aRR: 0.94; 95% CI: 0.89–0.98; p < 0.01), but not with the influenza type.

### Co-infection with respiratory viruses other than influenza among children hospitalised with SARI

Results for additional respiratory viruses, whose testing was implemented from season 2015/16 onwards, are listed in detail in Supplementary Table S8 and were available for 1,701 (72.8%) of the 2,336 patients with known status of complications (Figure 1). After reclassification of the infection status, taking into account the results for the non-influenza respiratory virus (NIRV) testing, 428 of those (25.2%) were defined as influenza-positive only, 838 (49.3%) as positive for NIRVs but influenza-negative and 143 (8.4%) as co-infected with influenza virus and additional NIRVs (Table 1).

**Table 1:** Age and length of stay of children < 15 years hospitalised with SARI and tested for multiple respiratory viruses by infection status, Belgium, 2015/16^a^–2019/20 (n = 1,701)

	Influenza virus only		Influenza virus plus co-infection		Non-influenza respiratory viruses		All negative	
	n	%	n	%	n	%	n	%
Total tested for additional respiratory viruses (n = 1,701)	428	25.2	143	8.4	838	49.3	292	17.2
Median age in years (IQR)	2.7 (1.0–4.9)		1.6 (0.8–3.0)		0.8 (0.3–1.8)		1.6 (0.4–4.9)	
Median length of hospital stay in days (IQR)	3.0 (2.0–5.0)		3.0 (2.0–4.0)		3.0 (2.0–5.0)		3.0 (2.0–5.0)	
Age groups (years)								
< 1 (n = 745)	107	14.4	46	6.2	471	63.2	121	16.2
1–4 (n = 721)	217	30.1	81	11.2	323	44.8	100	13.9
5–14 (n = 235)	104	44.3	16	6.8	44	18.7	71	30.2

SARI: severe acute respiratory infections.

^a^ Testing for other respiratory viruses was only implemented from season 2015/16 onwards.

Percentages calculated by row.

**Figure 3:**
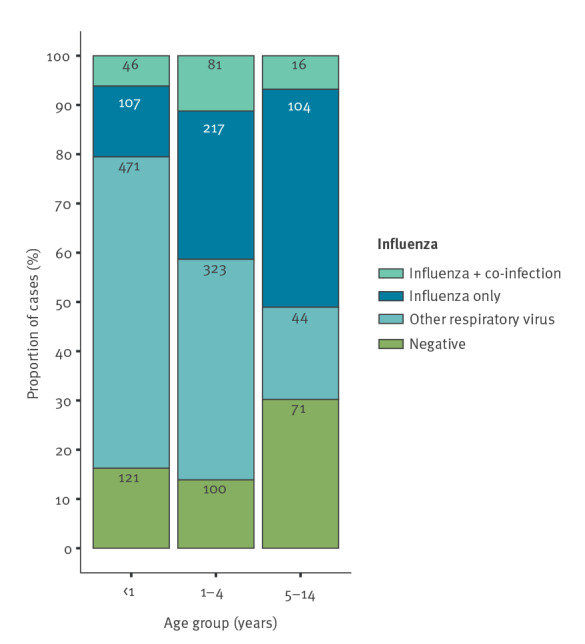
Multi-virus infection status among children < 15 years hospitalised with severe acute respiratory infections, Belgium, 2015/16^a^−2019/20 (n = 1,701)

^a^ Testing for other respiratory viruses was only implemented from season 2015/16 onwards.

One quarter of influenza-positive cases tested positive for an additional NIRV (25%; 143/571). Overall among the NIRV detected, picornaviruses (39.1%; 384/981), adenoviruses (25.7%; 252/981) and human metapneumoviruses (hMPV) (23.9%: 234/981) were the most common. Still, 17.2% (292/1,701) of cases remained negative for all tested viruses. The proportion of infections with NIRVs only was the highest in the youngest age group (63.2%; 471/745 for < 1 years) and decreased with increasing age (Table 1, Figure 3).

Numbers in black: total number of cases per category and age group.

The median age of cases infected with NIRVs only (0.8 years; IQR: 0.3–1.8) was lower than that of cases with influenza only (2.7 years; IQR: 1.0–4.9; Wilcoxon rank sum test: p < 0.0001; Table 1). Even if the medians were identical, the children positive for NIRVs only were found to have a significantly longer hospital stay than those positive for influenza only (Wilcoxon rank sum test: p < 0.01).

The proportion of complications (Table 2) in children infected with NIRVs only (33.7%; 282/838) was higher than in children with influenza virus infection only (21.7%; 93/428; proportions z-test: p < 0.0001) or co-infection with influenza virus (22.4%; 32/143; p = 0.01). The proportion of complications in children negative for any tested respiratory virus (28.8%; 84/292) was also higher than that in children with influenza virus infection only (proportions z-test: p = 0.04). There was no difference in complications between children with influenza virus infection only or with co-infection (Table 2). There was no difference in the proportion of patients with any risk factor or a single specific risk factor among the cases with complications in each infection status group.

**Table 2:** Complications among children < 15 years hospitalised with SARI and tested for multiple respiratory viruses, by infection status and risk factor, Belgium, 2015/16^a^–2019/20 (n = 1,701)

	Influenza virus only				Influenza virus plus co-infection				Non-influenza respiratory viruses				All negative			
	n	%	n	%	n	%	n	%	n	%	n	%	n	%	n	%
Complications	Yes		No		Yes		No		Yes		No		Yes		No	
Any complication	335	78.3	93	21.7	111	77.6	32	22.4	556	66.3	282	33.7	208	71.2	84	28.8
Any risk factor^b^																
Yes	23	6.9	21	22.6	8	7.2	9	28.1	51	9.2	53	18.8	29	13.9	18	21.4
No	261	77.9	65	69.9	91	82.0	22	68.8	450	80.9	191	67.7	143	68.8	49	58.3
Unknown	51	15.2	7	7.5	12	10.8	1	3.1	55	9.9	38	13.5	36	17.3	17	20.2
Type of risk factor among risk factor “yes”																
Asthma	6	26.1	10	47.6	2	25.0	4	44.4	16	31.4	22	41.5	8	27.6	9	50.0
Chronic respiratory disease	7	30.4	9	42.9	2	25.0	5	55.6	13	25.5	23	43.4	11	37.9	4	22.2
Neuromuscular disease	3	13.0	2	9.5	1	12.5	0	0.0	11	21.6	4	7.5	6	20.7	1	5.6
Immunodeficiency	6	26.1	0	0.0	1	12.5	0	0.0	6	11.8	2	3.8	5	17.2	2	11.1
Chronic cardiac disease	2	8.7	2	9.5	1	12.5	0	0.0	7	13.7	5	9.4	2	6.9	1	5.6
Diabetes	2	8.7	0	0.0	1	12.5	0	0.0	2	3.9	0	0.0	3	10.3	0	0.0
Renal insufficiency	2	8.7	0	0.0	0	0.0	0	0.0	4	7.8	1	1.9	0	0.0	1	5.6
Hepatic insufficiency	0	0.0	0	0.0	0	0.0	0	0.0	1	2.0	0	0.0	0	0.0	0	0.0
Obesity	0	0.0	1	4.8	0	0.0	0	0.0	1	2.0	1	1.9	1	3.4	0	0.0

SARI: severe acute respiratory infections.

^a^ Testing for other respiratory viruses was only implemented from season 2015/16 onwards.

^b^ Risk factor status among cases. Cases might have several risk factors.

Percentages calculated by column.

Multivariable modelling, using influenza-positive vs -negative test results as a classification for infection status, showed that being influenza-negative was associated with an increased risk of complications (aRR: 1.38; 95% CI: 1.18–1.61); for details see Supplementary Table S9. Using a model with the more detailed infection status defined by multi-virus testing and with influenza-positive as baseline (Model 1), children with NIRV infections had a 32% higher risk of complications (aRR: 1.32; 95% CI: 1.06–1.66; p < 0.01; Table 3)

**Table 3:** Univariate and multivariable analysis of risk of complications among children < 15 years hospitalised with severe acute respiratory infections, based on multi-virus testing, Belgium, 2015/16–2019/20

	No complication	Complication	Univariate RR (95% CI)	p value	Multivariable RR (95% CI)^a^	p value
Model 1 (n = 1,484)						
Infection status						
Influenza virus only	284	86	1	Reference		
Influenza virus + co-infection	99	31	1.03 (0.72–1.47)	ns	1.01 (0.69–1.42)	ns
Non-influenza respiratory viruses	501	244	1.41 (1.14–1.74)	0.001	1.32 (1.06–1.66)	0.01
Negative for any respiratory viruses tested	172	67	1.21 (0.92–1.59)	ns	1.10 (0.83–1.44)	ns
Risk factor^b^						
No	945	327	1			
Yes	111	101	1.85 (1.56–2.19)	< 0.001	1.88 (1.56–2.23)	< 0.0001
Sex						
Female	498	196	1	Reference		
Male	558	232	1.04 (0.89–1.22)	ns	1.05 (0.90–1.23)	ns
Patient age in years			1.00 (0.99–1.01)	ns	0.99 (0.96–1.02)	ns
Model 2 (n = 1,328)						
Infection status						
Influenza virus only	258	70	1	Reference		
Influenza virus + co-infection	86	27	1.12 (0.74–1.63)	ns	1.11 (0.74–1.61)	ns
Non-influenza respiratory viruses	410	238	1.72 (1.38–2.19)	< 0.0001	1.61 (1.27–2.07)	< 0.0001
Negative for any respiratory viruses tested	170	69	(1.35 (1.01–1.81)	ns	1.29 (0.97–1.72)	ns
Asthma						
No	895	365	1	Reference		
Yes	29	39	1.98 (1.55–2.42)	< 0.0001	1.87 (1.46–2.30)	< 0.0001
Sex						
Female	428	178	1			
Male	496	226	1.07 (0.91–1.26)	ns	1.04 (0.89–1.23)	ns
Patient age in years			0.98 (0.95–1.01)	ns	0.99 (0.96–1.02)	ns
Model 3 (n = 1,328)						
Infection status						
Influenza virus only	258	70	1	Reference		
Influenza virus + co-infection	86	27	1.12 (0.74–1.63)	ns	1.11 (0.74–1.61)	ns
Non-influenza respiratory viruses	410	238	1.72 (1.38–2.19)	< 0.0001	1.67 (1.32–2.14)	< 0.0001
Negative for any respiratory viruses tested	170	69	1.35 (1.01–1.81)	ns	1.28 (0.96–1.71)	ns
Chronic respiratory disease						
No	895	365	1	Reference		
Yes	29	39	1.88 (1.42–2.33)	< 0.0001	1.88 (1.44–2.32)	< 0.0001
Sex						
Female	428	178	1	Reference		
Male	496	226	1.07 (0.91–1.26)	ns	1.05 (0.90–1.24)	ns
Patient age in years			0.98 (0.95–1.01)	ns	0.99 (0.96–1.02)	ns

CI: confidence interval; ns: not significant; RR: risk ratio.

^a^ Multivariable analysis following principle of parsimony and adjusting for infection status, sex, risk factors and age in years.

^b^ Children with risk factor unknown were excluded from this analysis.

The risk of complications remained significantly associated with the presence of underlying risk factors (aRR: 1.88; 95% CI: 1.56–2.23; p < 0.0001; Table 3). When evaluating single risk factors, asthma (aRR: 1.87; 95% CI: 1.46–2.30; p < 0.0001; Model 2) and chronic respiratory disease (aRR: 1.88; 95% CI: 1.44–2.32; p < 0.0001; Model 3) were associated with complications. Testing negative for any respiratory virus tested was not associated with increased risk of complication.

## Discussion

Worldwide, acute respiratory infections are among the leading causes of death in children under 5 years [5]. In Belgium, we found that more than 80% of children hospitalised with SARI were younger than 5 years, but the overall mortality remained below 1%. The majority of children (ca 90%) had no risk factors for severe disease, but the proportion of children with risk factors increased with age. Complications occurred in about one third of cases and were associated with the presence of risk factors, specifically asthma or chronic respiratory disease, in agreement with the fact that viral lower respiratory tract infections were shown to be associated with exacerbation of acute asthma in children admitted to hospital [17].

Only one third of Belgian children with SARI tested positive for influenza, with a 3:1 ratio of type A to B, equivalent to studies in Finland and Canada spanning a similar time period [18,19]. Most seasons between 2011 and 2020 in Belgium were dominated by influenza type A, and children infected with type A tended to be younger than children infected with type B, as reported by others [19,20]. We noticed that the positivity for influenza increased with increasing age.

Around one quarter of influenza-positive children experienced complications, without clear difference between influenza types. Our study population was not sufficiently large for a detailed model on complications by subtype or lineage. Increased severity for the 2009 pandemic H1N1 subtype has been described for children in Ireland [21]. However, a recent literature review including 47 studies across all ages concluded that virus subtype or lineage was not associated with severity [22]. Although the proportion of risk factors was the same in influenza-positive or -negative children, complications among influenza-positive children were associated with underlying risk factors in our study. However, the number of influenza-positive cases was insufficient to assess the impact of specific risk factors. Others have reported association of chronic pulmonary disease, asthma, cardiac disease and neurological or neuromuscular disease with risk of hospitalisation in children with influenza in Norway, and with increased probability of respiratory failure in children hospitalised with influenza in the United States [23,24].

Multi-virus testing enabled the detection of NIRVs in more than half of Belgian children hospitalised for SARI, with prevalence being the highest in the youngest age group and decreasing in older age groups. The most common NIRVs were picornaviruses, adenoviruses and hMPV. A recent study on acute respiratory infections in children in South America found RSV among the most common in the age group younger than 2 years in need of hospitalisation [25]. However, the annual sampling periods in our study did not coincide with the RSV season in Belgium [26]. More than 15% of specimens remained negative for all tested respiratory viruses. This proportion nearly doubled in children 5–14 years. Other aetiologies including bacteria should therefore not be neglected. In a study in Niger, *Streptococcus pneumoniae* was identified in 56% and *Haemophilus influenzae* type b in 12% of influenza-negative SARI cases in children younger than 5 years [27].

Integration of multi-virus testing results showed that infection with NIRVs alone might lead to a higher risk of complication compared with infection with influenza viruses in children. In concordance with the three most common NIRVs observed in our population, a study conducted from 2000 to 2002 in Finland found that respiratory picornaviruses were associated with hospitalisation for bronchiolitis and acute asthma in 65% of children aged 1–2 years and 82% of children older than 3 years [28]. A hospital-based surveillance study in children in Vietnam found that hMPV infections had 1.5-fold higher odds of being severe whereas adenovirus infections consistently had a lower risk of severity [4]. In light of overlapping symptoms with influenza, the burden of NIRV infections in children might be generally underestimated due to lack of testing.

Vaccines targeting NIRVs are currently not available, but development of vaccines for RSV, respiratory picornaviruses and hMPV are advancing [11-13]. A birth cohort study conducted in Scotland between 2007 and 2015 found that children under 6 months of age were at increased risk of hospitalisation due to influenza if they had older siblings [29]. In our study, we saw the highest proportion of influenza positivity in children 5–14 years, where the overall rate of vaccination with the influenza vaccine available during the relevant season was 14.4%. Targeting those older children for influenza vaccination programmes may help protect their younger siblings against severe influenza, as influenza vaccination of children younger than 6 months is currently not recommended in Belgium. Conversely, maternal influenza vaccination has been shown to decrease the risk of infant hospitalisation due to infection with influenza viruses, but also NIRVs early in life, and is now recommended in most European countries including Belgium [30]. Unfortunately, the vaccination status of mothers is currently not recorded in the Belgian surveillance system.

The main limitation of our study is that the current surveillance is only performed during the winter months and therefore does not capture influenza virus circulation during the rest of the year, nor the epidemiology of NIRVs outside the influenza season. There is currently no specific paediatric surveillance network in place, which hinders the comparison of prevalence of respiratory viruses in hospitalised children vs the population served by general practitioners. Our sample size prevented a robust analysis of single risk factors and of the association of influenza virus subtypes or lineages with complications, as well as the severity of each NIRV separately. Therefore, continued surveillance, including influenza virus typing and subtyping and integrated surveillance of NIRVs, needs to be maintained and expanded in time and scope, as recommended by the European Centre for Disease Control and Prevention (ECDC) and the WHO [31,32]. As specimens are currently not routinely tested for the presence of bacteria by the NIC, we cannot draw any conclusions about cases negative for all tested respiratory viruses, or about viral and bacterial co-infection in our population. With the limited data on influenza vaccination coverage, the assessment of the effect of vaccination on the occurrence of complications was beyond the scope of this study. The arrival of new vaccines against NIRVs (e.g. RSV) should be associated with an overall improvement of the registration of vaccination data.

## Conclusion

We showed that the presence of underlying risk factors, especially asthma and chronic respiratory disease, is associated with a higher risk of complications in children with SARI, regardless of patient age or detailed infection status. Children with underlying risk factors should thus receive priority care when admitted with SARI, no matter the aetiology. Following the COVID-19 pandemic, a comprehensive year-round surveillance based on sentinel systems for the assessment of influenza viruses, severe acute respiratory syndrome coronavirus 2 (SARS-CoV-2), and potentially NIRVs, is a public health priority to accurately follow virus-specific disease incidence and severity and to assess vaccine effectiveness. Inclusion of multiplex PCR testing strategies for other common respiratory viruses is valuable to distinguish the true burden of disease attributable to seasonal influenza. We showed that, in children, NIRVs are responsible for approximately half of the cases in need of hospitalisation, and that the risk of complications is higher for those infected with NIRV infections compared with influenza, whatever the risk factor status or age. There is thus a clear need for diagnostic and prevention strategies for NIRVs targeted at children and/or pregnant women, as they could have a substantial effect on the burden of respiratory infections in children. 

## Supplementary Data

392000 Supplement

## References

[1] Krammer F Smith GJD Fouchier RAM Peiris M Kedzierska K Doherty PC Influenza. Nat Rev Dis Primers. 2018;4(1):3. 10.1038/s41572-018-0002-y 29955068 PMC7097467

[2] Martínez A Soldevila N Romero-Tamarit A Torner N Godoy P Rius C Risk factors associated with severe outcomes in adult hospitalized patients according to influenza type and subtype. PLoS One. 2019;14(1):e0210353. 10.1371/journal.pone.0210353 30633778 PMC6329503

[3] Mandelia Y Procop GW Richter SS Worley S Liu W Esper F . Dynamics and predisposition of respiratory viral co-infections in children and adults. Clin Microbiol Infect. 2021;27(4):631.e1-6. 10.1016/j.cmi.2020.05.042 32540470

[4] Althouse BM Flasche S Toizumi M Nguyen HT Vo HM Le MN Differences in clinical severity of respiratory viral infections in hospitalized children. Sci Rep. 2021;11(1):5163. 10.1038/s41598-021-84423-2 33664311 PMC7933285

[5] World Health Organisation (WHO). World health statistics 2023: monitoring health for the SDGs, sustainable Development goals. Geneva: WHO; 2023. Available from: https://www.who.int/publications/i/item/9789240074323

[6] World Health Organisation (WHO). Global influenza strategy summary 2019-2030 influenza. Geneva: WHO; 2019. Available from: https://www.who.int/publications/i/item/9789241515320

[7] Iuliano AD Roguski KM Chang HH Muscatello DJ Palekar R Tempia S Estimates of global seasonal influenza-associated respiratory mortality: a modelling study. Lancet. 2018;391(10127):1285-300. 10.1016/S0140-6736(17)33293-2 29248255 PMC5935243

[8] Wang X Li Y O’Brien KL Madhi SA Widdowson MA Byass P Global burden of respiratory infections associated with seasonal influenza in children under 5 years in 2018: a systematic review and modelling study. Lancet Glob Health. 2020;8(4):e497-510. 10.1016/S2214-109X(19)30545-5 32087815 PMC7083228

[9] World Health Organization . Vaccines against influenza: WHO position paper – May 2022. Wkly Epidemiol Rec. 2022;97(19):185-208.

[10] Conseil Supérieur de la Santé. Avis du conseil supérieur de la santé n° 9488. Vaccination contre la grippe saisonnière. Saison hivernale 2018-2019. [Opinion of the superior health council no. 9488. Seasonal influenza vaccination. Winter season 2018/19]. Bruxelles: Conseil Supérieur de la Santé; 2018. French. Available from: https://www.health.belgium.be/sites/default/files/uploads/fields/fpshealth_theme_file/css_9488_avis_grippe_update201810.pdf

[11] Williams K Bastian AR Feldman RA Omoruyi E de Paepe E Hendriks J Phase 1 safety and immunogenicity study of a respiratory syncytial virus vaccine with an adenovirus 26 vector encoding prefusion F (Ad26.RSV.preF) in adults aged ≥60 Years. J Infect Dis. 2020;222(6):979-88. 10.1093/infdis/jiaa193 32320465

[12] Karron RA San Mateo J Wanionek K Collins PL Buchholz UJ . Evaluation of a live attenuated human metapneumovirus vaccine in adults and children. J Pediatric Infect Dis Soc. 2018;7(1):86-9. 10.1093/jpids/pix006 28444226 PMC6075531

[13] Edlmayr J Niespodziana K Popow-Kraupp T Krzyzanek V Focke-Tejkl M Blaas D Antibodies induced with recombinant VP1 from human rhinovirus exhibit cross-neutralisation. Eur Respir J. 2011;37(1):44-52. 10.1183/09031936.00149109 20530036

[14] Bossuyt N, Thomas I, Barbezange C, Bustos-Sierra N, Van Cauteren D, Vermeulen M. Surveillance des infections à influenza: rapport épidémiologique, saison 2018-2019 [Surveillance of influenza infections : epidemiological report, 2018-2019 season]. Brussels: Sciensano; 2019. D/2019/14.440/103. Available from: https://www.sciensano.be/en/biblio/surveillance-des-infections-a-influenza-rapport-epidemiologique-saison-2018-2019

[15] Fischer N Dauby N Bossuyt N Reynders M Gérard M Lacor P Monitoring of human coronaviruses in Belgian primary care and hospitals, 2015-20: a surveillance study. Lancet Microbe. 2021;2(3):e105-14. 10.1016/S2666-5247(20)30221-4 33937883 PMC8064766

[16] R Core Team. R: A language and environment for statistical computing. Vienna: R Foundation for Statistical Computing. [Accessed: 18 Mar 2022]. Available from: https://www.r-project.org

[17] Kwon JM Shim JW Kim DS Jung HL Park MS Shim JY . Prevalence of respiratory viral infection in children hospitalized for acute lower respiratory tract diseases, and association of rhinovirus and influenza virus with asthma exacerbations. Korean J Pediatr. 2014;57(1):29-34. 10.3345/kjp.2014.57.1.29 24578714 PMC3935110

[18] Mattila JM Vuorinen T Heikkinen T . Comparative severity of influenza A and B infections in hospitalized children. Pediatr Infect Dis J. 2020;39(6):489-93. 10.1097/INF.0000000000002610 32091502

[19] Tran D Vaudry W Moore D Bettinger JA Halperin SA Scheifele DW Hospitalization for Influenza A Versus B. Pediatrics. 2016;138(3):e20154643. 10.1542/peds.2015-4643 27535144

[20] Peltola V Ziegler T Ruuskanen O . Influenza A and B virus infections in children. Clin Infect Dis. 2003;36(3):299-305. 10.1086/345909 12539071

[21] Rebolledo J Igoe D O’Donnell J Domegan L Boland M Freyne B Influenza in hospitalized children in Ireland in the pandemic period and the 2010/2011 season: risk factors for paediatric intensive-care-unit admission. Epidemiol Infect. 2014;142(9):1826-35. 10.1017/S0950268813002732 24229618 PMC9151236

[22] Caini S Kroneman M Wiegers T El Guerche-Séblain C Paget J . Clinical characteristics and severity of influenza infections by virus type, subtype, and lineage: A systematic literature review. Influenza Other Respir Viruses. 2018;12(6):780-92. 10.1111/irv.12575 29858537 PMC6185883

[23] Hauge SH Bakken IJ de Blasio BF Håberg SE . Risk conditions in children hospitalized with influenza in Norway, 2017-2019. BMC Infect Dis. 2020;20(1):769. 10.1186/s12879-020-05486-6 33076855 PMC7569759

[24] Keren R Zaoutis TE Bridges CB Herrera G Watson BM Wheeler AB Neurological and neuromuscular disease as a risk factor for respiratory failure in children hospitalized with influenza infection. JAMA. 2005;294(17):2188-94. 10.1001/jama.294.17.2188 16264160

[25] Azziz-Baumgartner E Duca LM González R Calvo A Kaydos-Daniels SC Olson N Incidence of respiratory virus illness and hospitalizations in a Panama and El Salvador birth cohort, 2014-2018. Lancet Reg Health Am. 2022;13:100304. 10.1016/j.lana.2022.100304 36189114 PMC9485193

[26] Subissi L Bossuyt N Reynders M Gérard M Dauby N Bourgeois M Capturing respiratory syncytial virus season in Belgium using the influenza severe acute respiratory infection surveillance network, season 2018/19. Euro Surveill. 2020;25(39):1900627. 10.2807/1560-7917.ES.2020.25.39.1900627 33006303 PMC7531071

[27] Lagare A Maïnassara HB Issaka B Sidiki A Tempia S . Viral and bacterial etiology of severe acute respiratory illness among children < 5 years of age without influenza in Niger. BMC Infect Dis. 2015;15(1):515. 10.1186/s12879-015-1251-y 26567015 PMC4644278

[28] Jartti T Lehtinen P Vuorinen T Osterback R van den Hoogen B Osterhaus AD Respiratory picornaviruses and respiratory syncytial virus as causative agents of acute expiratory wheezing in children. Emerg Infect Dis. 2004;10(6):1095-101. 10.3201/eid1006.030629 15207063 PMC3323183

[29] Hardelid P Verfuerden M McMenamin J Gilbert R . Risk factors for admission to hospital with laboratory-confirmed influenza in young children: birth cohort study. Eur Respir J. 2017;50(3):1700489. 10.1183/13993003.00489-2017 28954782

[30] Zaman K Roy E Arifeen SE Rahman M Raqib R Wilson E Effectiveness of maternal influenza immunization in mothers and infants. N Engl J Med. 2008;359(15):1555-64. 10.1056/NEJMoa0708630 18799552

[31] World Health Organisation (WHO). End-to-end integration of SARS-CoV-2 and influenza sentinel surveillance: revised interim guidance. Geneva: WHO; 2022. Available from: https://www.who.int/publications/i/item/WHO-2019-nCoV-Integrated_sentinel_surveillance-2022.1

[32] World Health Organisation Regional Office for Europe (WHO/Europe), European Centre for Disease Prevention and Control (ECDC). Operational considerations for respiratory virus surveillance in Europe. Copenhagen: WHO/Europe and Stockholm: ECDC; 2022. Available from: https://www.ecdc.europa.eu/en/publications-data/operational-considerations-respiratory-virus-surveillance-europe

