# Supplementary material for: Influenza versus other respiratory viruses – assessing severity among hospitalised children, Belgium, 2011 to 2020
Source: Euro Surveill. 2023 Jul 20;28(29):2300056. doi: 10.2807/1560-7917.ES.2023.28.29.2300056 (PMC10360368; doi:10.2807/1560-7917.ES.2023.28.29.2300056)
Supplement: Supplement [file 23-00056_BARBEZANGE_Supplement.pdf]

Supplementary Material

This supplementary material is hosted by Eurosurveillance as supporting information alongside the article **Influenza versus other respiratory viruses – Assessing severity among hospitalized children in Belgium (2011-2020)**, on behalf of the authors, who remain responsible for the accuracy and appropriateness of the content. The same standards for ethics, copyright, attributions and permissions as for the article apply. Supplements are not edited by Eurosurveillance and the journal is not responsible for the maintenance of any links or email addresses provided therein.

TABLE OF CONTENTS

1 SUPPLEMENTARY TABLES: 2

1.1 Supplementary Table S1: Summary table of characteristics of children <15 y/o hospitalized with SARI in Belgium during influenza seasons 2011-2012 to 2019-2020....2
1.2 Supplementary Table S2: Underlying risk factors among children <15 y/o hospitalized with SARI in Belgium during influenza seasons 2011-2012 to 2019-2020, per sex and age group. 3
1.3 Supplementary Table S3: Influenza infections among children <15 y/o hospitalized with SARI in Belgium during influenza seasons 2011-2012 to 2019-2020, by type and subtype/lineage. 4
1.4 Supplementary Table S4: Underlying risk factors among children <15 y/o hospitalized with SARI in Belgium during influenza seasons 2011-2012 to 2019-2020, per influenza type, subtype and lineage. 5
1.5 Supplementary Table S5: Occurrence of complications among children <15 y/o hospitalized with SARI in Belgium during influenza seasons 2011-2012 to 2019-2020....6
1.6 Supplementary Table S6: Occurrence of complications among children <15 y/o hospitalized with SARI in Belgium during influenza seasons 2011-2012 to 2019-2020, per influenza type, subtype and lineage. 7
1.7 Supplementary Table S7: Univariate and Multivariable analysis of the risk for complications among influenza positive children <15 y/o hospitalized with SARI in Belgium during influenza seasons 2011-2012 to 2019-2020....8
1.8 Supplementary Table S8: Identified respiratory viruses in children hospitalized with SARI in Belgium during influenza seasons 2015-2016 to 2019-2020. 9
1.9 Supplementary Table S9: Univariate and Multivariable analysis of the risk for complications among influenza-positive and -negative children <15 y/o hospitalized with SARI in Belgium during influenza seasons 2011-2012 to 2019-2020. 10

2 SUPPLEMENTARY METHODS 11

2.1 Study design and data collection 11
2.2 Laboratory testing....11
2.3 Study population 11
2.4 data analysis tools and r packages....11

3 SUPPLEMENTARY REFERENCES....11

1 SUPPLEMENTARY TABLES:

1.1 SUPPLEMENTARY TABLE S1: SUMMARY TABLE OF CHARACTERISTICS OF CHILDREN <15 Y/O HOSPITALIZED WITH SARI IN BELGIUM DURING INFLUENZA SEASONS 2011-2012 TO 2019-2020.

**Total**	**All ages (N = 2,944)**	**<1 y/o (N = 1,279)**	**1-4 y/o (N = 1,252)**	**5-14 y/o (N = 413)**
**Sex**				
Female	1,327 (45.1%)	542 (42.4%)	586 (46.8%)	199 (48.2%)
Male	1,617 (54.9%)	737 (57.6%)	666 (53.2%)	214 (51.8%)
**Patient Age Group**				
<1 y/o	1,279 (43.5%)	-	-	-
1-4 y/o	1,252 (42.5%)	-	-	-
5-14 y/o	413 (14.0%)	-	-	-
**Median (IQR)**	1.2 (0.4, 3.1)	0.3 (0.2, 0.6)	2.1 (1.4, 3.1)	7.6 (6.0, 10)
**Hospital Region**				
Brussels	1,429 (48.5%)	702 (54.9%)	551 (44.0%)	176 (42.6%)
Flanders	907 (30.8%)	304 (23.8%)	444 (35.5%)	159 (38.5%)
Wallonia	608 (20.7%)	273 (21.3%)	257 (20.5%)	78 (18.9%)
**Flu vaccine**				
Information available	2515 (85.4%)	1169 (91.4%)	1005 (80.3%)	341 (82.6%)
Yes (among information known)	111 (4.4%)	16 (1.4%)	46 (4.6%)	49 (14.4%)
No (among information known)	2,404 (95.6%)	1,153 (98.6%)	959 (95.4%)	292 (85.6%)
Information missing	429 (14.6%)	110 (8.6%)	247 (19.7%)	72 (17.4%)
**Anti-viral treatment**				
Information available	2442 (82.9%)	1084 (84.8%)	1027 (82.0%)	331 (80.1%)
Yes (among information known)	45 (1.8%)	16 (1.5%)	19 (1.9%)	10 (3.0%)
No (among information known)	2,397 (98.2%)	1,068 (98.5%)	1,008 (98.1%)	321 (97.0%)
Information missing	502 (17.1%)	195 (15.2%)	225 (18.0%)	82 (19.9%)
**Antibiotic treatment**				
Information available	1723 (58.5%)	795 (62.1%)	687 (54.9%)	241 (58.4%)
Yes (among information known)	704 (40.9%)	287 (36.1%)	312 (45.4%)	105 (43.6%)
No (among information known)	1,019 (59.1%)	508 (63.9%)	375 (54.6%)	136 (56.4%)
Information missing	1,221 (41.5%)	484 (37.9%)	565 (45.1%)	172 (41.6%)

SARI: severe acute respiratory infection, y/o: years old, IQR: Interquartile range.

1.2 SUPPLEMENTARY TABLE S2: UNDERLYING RISK FACTORS AMONG CHILDREN <15 Y/O HOSPITALIZED WITH SARI IN BELGIUM DURING INFLUENZA SEASONS 2011-2012 TO 2019-2020, PER SEX AND AGE GROUP.

	**Total (N = 2,944)**	**Female (N = 1,327)**	**Male (N = 1,617)**	**<1 y/o (N = 1,279)**	**1-4 y/o (N = 1,252)**	**5-14 y/o (N = 413)**
**Any risk factor**						
Unknown	461 (15.7%)	185 (13.9%)	276 (17.1%)	214 (16.7%)	188 (15.0%)	59 (14.3%)
Yes	359 (12.2%)	175 (13.2%)	184 (11.4%)	74 (5.8%)	160 (12.8%)	125 (30.3%)
No	2,124 (72.1%)	967 (72.9%)	1,157 (71.6%)	991 (77.5%)	904 (72.2%)	229 (55.4%)
**Nr of risk factors among children with risk factors**						
One	321 (89.4%)	155 (88.6%)	166 (90.2%)	66 (89.2%)	148 (92.5%)	107 (85.6%)
Two	34 (9.5%)	19 (10.9%)	15 (8.2%)	8 (10.8%)	12 (7.5%)	14 (11.2%)
Three or more	4 (1.2%)	1 (0.5%)	3 (1.6%)	0 (0.0%)	0 (0.0%)	4 (3.2%)
**Type of risk factor***						
Asthma	130 (5.3%)	62 (5.6%)	68 (5.0%)	9 (0.8%)	65 (6.2%)	56 (15.9%)
Chronic respiratory disease	112 (4.6%)	58 (5.3%)	54 (4.0%)	31 (2.9%)	47 (4.5%)	34 (9.8%)
Neuromuscular disease	49 (2.0%)	21 (1.9%)	28 (2.1%)	9 (0.8%)	23 (2.2%)	17 (4.9%)
Immunodeficiency	42 (1.7%)	21 (1.9%)	21 (1.6%)	10 (0.9%)	17 (1.6%)	15 (4.3%)
Chronic cardiac disease	43 (1.8%)	22 (2.0%)	21 (1.6%)	13 (1.2%)	16 (1.5%)	14 (4.0%)
Diabetes	8 (0.3%)	5 (0.5%)	3 (0.2%)	0 (0.0%)	3 (0.3%)	5 (1.4%)
Renal insufficiency	10 (0.4%)	4 (0.4%)	6 (0.4%)	7 (0.7%)	0 (0.0%)	3 (0.9%)
Hepatic insufficiency	1 (0.0%)	1 (0.1%)	0 (0.0%)	1 (0.1%)	0 (0.0%)	0 (0.0%)
Obesity	6 (0.2%)	2 (0.2%)	4 (0.3%)	2 (0.2%)	1 (0.1%)	3 (0.9%)
**Outcome known**	2,942 (99.9%)	1,326 (99.9%)	1,616 (99.9%)	1,278 (99.9%)	1,252 (100%)	412 (99.8%)
Alive	2,935 (99.8%)	1,323 (99.8%)	1,612 (99.8%)	1,273 (99.6%)	1,250 (99.8%)	412 (100%)
Dead	7 (0.2%)	3 (0.2%)	4 (0.2%)	5 (0.4%)	2 (0.2%)	0 (0.0%)

*Multiple risk factors can occur per case.
SARI: severe acute respiratory infection, y/o: years old.

1.3 Supplementary Table S3: Influenza infections among children <15 y/o hospitalized with SARI in Belgium during influenza seasons 2011-2012 to 2019-2020, by type and subtype/lineage.

	Total (N = 2,944)	Influenza negative	Influenza positive	Type A amongst positive	Type B amongst positive	H1N1 amongst type A	H3N2 amongst type A	Unidentified type A	Victoria amongst type B	Yamagata amongst type B	Unidentified lineage B
**Total (N = 2,944)**		2,006 (68.1%)	938 (31.9%)	701 (74.7%)	237 (25.3%)	419 (59.8%)	257 (36.7%)	25 (3.6%)	100 (42.2%)	129 (54.4%)	8 (3.4%)
**Season**											
2011/2012 (N = 70)	70 (2.4%)	52 (74.3%)	18 (25.7%)	17 (94.4%)	1 (5.6%)	0 (0.0%)	15 (88.2%)	2 (11.8%)	0 (0.0%)	0 (0.0%)	1 (100.0%)
2012/2013 (N = 257)	257 (8.7%)	153 (59.5%)	104 (40.5%)	50 (48.1%)	54 (51.9%)	45 (90.0%)	4 (8.0%)	1 (2.0%)	1 (1.9%)	50 (92.6%)	3 (5.6%)
2013/2014 (N = 173)	173 (5.9%)	138 (79.8%)	35 (20.2%)	35 (100.0%)	0 (0.0%)	14 (40.0%)	17 (48.6%)	4 (11.4%)	0 (0.0%)	0 (0.0%)	0 (0.0%)
2014/2015 (N = 307)	307 (10.4%)	226 (73.6%)	81 (26.4%)	75 (92.6%)	6 (7.4%)	18 (24.0%)	50 (66.7%)	7 (9.3%)	0 (0.0%)	6 (100.0%)	0 (0.0%)
2015/2016 (N = 352)	352 (12.0%)	191 (54.3%)	161 (45.7%)	86 (53.4%)	75 (46.6%)	85 (98.8%)	1 (1.2%)	0 (0.0%)	73 (97.3%)	0 (0.0%)	2 (2.7%)
2016/2017 (N = 247)	247 (8.4%)	199 (80.6%)	48 (19.4%)	47 (97.9%)	1 (2.1%)	0 (0.0%)	43 (91.5%)	4 (8.5%)	1 (100.0%)	0 (0.0%)	0 (0.0%)
2017/2018 (N = 556)	556 (18.9%)	348 (62.6%)	208 (37.4%)	133 (63.9%)	75 (36.1%)	120 (90.2%)	12 (9.0%)	1 (0.8%)	1 (1.3%)	72 (96.0%)	2 (2.7%)
2018/2019 (N = 482)	482 (16.4%)	364 (75.5%)	118 (24.5%)	116 (98.3%)	2 (1.7%)	45 (38.8%)	70 (60.3%)	1 (0.9%)	1 (50.0%)	1 (50.0%)	0 (0.0%)
2019/2020 (N = 500)	500 (17.0%)	335 (67.0%)	165 (33.0%)	142 (86.1%)	23 (13.9%)	92 (64.8%)	45 (31.7%)	5 (3.5%)	23 (100.0%)	0 (0.0%)	0 (0.0%)
**Patient Sex**											
Female (N = 1327)	1,327 (45.1%)	869 (65.5%)	458 (34.5%)	336 (73.4%)	122 (26.6%)	201 (59.8%)	125 (37.2%)	10 (3.0%)	47 (38.5%)	70 (57.4%)	5 (4.1%)
Male (N = 1617)	1,617 (54.9%)	1,137 (70.3%)	480 (29.7%)	365 (76.0%)	115 (24.0%)	218 (59.7%)	132 (36.2%)	15 (4.1%)	53 (46.1%)	59 (51.3%)	3 (2.6%)
**Age Group**											
<1 y/o (N = 1279)	1,279 (43.4%)	1,016 (79.4%)	263 (20.6%)	214 (81.4%)	49 (18.6%)	127 (59.3%)	81 (37.9%)	6 (2.8%)	18 (36.7%)	29 (59.2%)	2 (4.1%)
1-4 y/o (N = 1252)	1,252 (42.5%)	783 (62.5%)	469 (37.5%)	368 (78.5%)	101 (21.5%)	230 (62.5%)	122 (33.2%)	16 (4.3%)	52 (51.5%)	44 (43.6%)	5 (5.0%)
5-14 y/o (N = 413)	413 (14.0%)	207 (50.1%)	206 (49.9%)	119 (57.8%)	87 (42.2%)	62 (52.1%)	54 (45.4%)	3 (2.5%)	30 (34.5%)	56 (64.4%)	1 (1.1%)
**Median age in years (IQR)**	1.2 (0.4, 3.1)	1.0 (0.3, 2.4)	2.2 (0.8, 4.5)	2.0 (0.7, 3.9)	3.7 (1.3, 6.1)	1.9 (0.7, 3.7)	2.0 (0.5, 4.0)	1.9 (1.0, 4.0)	3.3 (1.5, 5.7)	4.0 (1.3, 7.0)	1.4 (0.9, 2.7)
**Median length of hospital stay in days (IQR)**	3.0 (2.0, 5.0)	3.0 (2.0, 5.0)	3.0 (2.0, 5.0)	3.0 (2.0, 5.0)	3.0 (2.0, 4.0)	3.0 (2.0, 5.0)	3.0 (2.0, 4.0)	3.0 (2.0, 3.0)	3.0 (2.0, 4.0)	3.0 (2.0, 4.0)	2.0 (1.0, 2.2)

*Multiple risk factors can occur per case.

SARI: severe acute respiratory infection, y/o: years old, IQR: Interquartile range.

1.4 Supplementary Table S4: Underlying risk factors among children <15 y/o hospitalized with SARI in Belgium during influenza seasons 2011-2012 to 2019-2020, per influenza type, subtype and lineage.

Total (N = 2,944)	Influenza negative (N = 2,006)	Influenza positive (N = 938)	Type A amongst positive (N = 701)	Type B amongst positive (N = 237)	H1N1 amongst Type A (N = 419)	H3N2 amongst Type A (N = 257)	Unidentified type A (N = 25)	Victoria amongst Type B (N = 100)	Yamagata amongst Type B (N = 129)	Unidentified lineage B (N = 8)
**Any risk factor***										
No (N = 2,124)	1,431 (67.4%)	693 (32.6%)	525 (75.8%)	168 (24.2%)	317 (60.4%)	190 (36.2%)	18 (3.4%)	72 (42.9%)	90 (53.6%)	6 (3.6%)
Yes (N = 359)	252 (70.2%)	107 (29.8%)	78 (72.9%)	29 (27.1%)	42 (53.8%)	31 (39.7%)	5 (6.4%)	6 (20.7%)	22 (75.9%)	1 (3.4%)
Unknown (N = 461)	323 (70.1%)	138 (29.9%)	98 (71.0%)	40 (29.0%)	60 (61.2%)	36 (36.7%)	2 (2.0%)	22 (55.0%)	17 (42.5%)	1 (2.5%)
**Type of risk factor**										
**Asthma**										
No (N = 2,329)	1,582 (67.9%)	747 (32.1%)	541 (72.4%)	206 (27.6%)	318 (58.8%)	204 (37.7%)	19 (3.5%)	74 (35.9%)	124 (60.2%)	8 (3.9%)
Yes (N = 130)	96 (73.8%)	34 (26.2%)	26 (76.5%)	8 (23.5%)	13 (50.0%)	11 (42.3%)	2 (7.7%)	3 (37.5%)	5 (62.5%)	0 (0.0%)
**Chronic respiratory disease**										
No (N = 2,345)	1,607 (68.5%)	738 (31.5%)	535 (72.5%)	203 (27.5%)	312 (58.3%)	204 (38.1%)	19 (3.6%)	75 (36.9%)	120 (59.1%)	8 (3.9%)
Yes (N = 112)	74 (66.1%)	38 (33.9%)	27 (71.1%)	11 (28.9%)	17 (63.0%)	9 (33.3%)	1 (3.7%)	2 (18.2%)	9 (81.8%)	0 (0.0%)
**Neuromuscular disease**										
No (N = 2,406)	1,645 (68.4%)	761 (31.6%)	555 (72.9%)	206 (27.1%)	324 (58.4%)	211 (38.0%)	20 (3.6%)	75 (36.4%)	123 (59.7%)	8 (3.9%)
Yes (N = 49)	36 (73.5%)	13 (26.5%)	5 (38.5%)	8 (61.5%)	3 (60.0%)	2 (40.0%)	0 (0.0%)	2 (25.0%)	6 (75.0%)	0 (0.0%)
**Immunodeficiency**										
No (N = 2,412)	1,650 (68.4%)	762 (31.6%)	552 (72.4%)	210 (27.6%)	323 (58.5%)	210 (38.0%)	19 (3.4%)	76 (36.2%)	127 (60.5%)	7 (3.3%)
Yes (N = 42)	27 (64.3%)	15 (35.7%)	11 (73.3%)	4 (26.7%)	6 (54.5%)	4 (36.4%)	1 (9.1%)	1 (25.0%)	2 (50.0%)	1 (25.0%)
**Chronic cardiac disease**										
No (N = 2,409)	1,647 (68.4%)	762 (31.6%)	548 (71.9%)	214 (28.1%)	322 (58.8%)	207 (37.8%)	19 (3.5%)	77 (36.0%)	129 (60.3%)	8 (3.7%)
Yes (N = 43)	30 (69.8%)	13 (30.2%)	13 (100.0%)	0 (0.0%)	7 (53.8%)	5 (38.5%)	1 (7.7%)	0 (0%)	0 (0%)	0 (0%)
**Diabetes**										
No (N = 2,440)	1,668 (68.4%)	772 (31.6%)	559 (72.4%)	213 (27.6%)	327 (58.5%)	212 (37.9%)	20 (3.6%)	77 (36.2%)	128 (60.1%)	8 (3.8%)
Yes (N = 8)	5 (62.5%)	3 (37.5%)	2 (66.7%)	1 (33.3%)	1 (50.0%)	1 (50.0%)	0 (0.0%)	0 (0.0%)	1 (100.0%)	0 (0.0%)
**Renal insufficiency**										
No (N = 2,435)	1,664 (68.3%)	771 (31.7%)	558 (72.4%)	213 (27.6%)	327 (58.6%)	211 (37.8%)	20 (3.6%)	77 (36.2%)	128 (60.1%)	8 (3.8%)
Yes (N = 10)	8 (80.0%)	2 (20.0%)	1 (50.0%)	1 (50.0%)	0 (0.0%)	1 (100.0%)	0 (0.0%)	0 (0.0%)	1 (100.0%)	0 (0.0%)
**Hepatic insufficiency**										
No (N = 2,443)	1,670 (68.4%)	773 (31.6%)	559 (72.3%)	214 (27.7%)	327 (58.5%)	212 (37.9%)	20 (3.6%)	77 (36.0%)	129 (60.3%)	8 (3.7%)
Yes (N = 1)	1 (100.0%)	0 (0.0%)	0 (0%)	0 (0%)	0 (0%)	0 (0%)	0 (0%)	0 (0%)	0 (0%)	0 (0%)
**Obesity**										
No (N = 2,438)	1,666 (68.3%)	772 (31.7%)	558 (72.3%)	214 (27.7%)	326 (58.4%)	212 (38.0%)	20 (3.6%)	77 (36.0%)	129 (60.3%)	8 (3.7%)
Yes (N = 6)	5 (83.3%)	1 (16.7%)	1 (100.0%)	0 (0.0%)	1 (100.0%)	0 (0.0%)	0 (0.0%)	0 (0%)	0 (0%)	0 (0%)

* Cases might have several risk factors.
SARI: severe acute respiratory infection, y/o: years old.

1.5 SUPPLEMENTARY TABLE S5: OCCURRENCE OF COMPLICATIONS AMONG CHILDREN <15 Y/O HOSPITALIZED WITH SARI IN BELGIUM DURING INFLUENZA SEASONS 2011-2012 TO 2019-2020.

	Any complication		% among any complication "yes"			
	**No**	**Yes***	**Pneumonia**	**Respiratory Assistance**	**ICU**	**ARDS**
Total (N = 2,336)**	1,602 (68.6%)	734 (31.4%)	565 (77.3%)	185 (25.2%)	56 (7.6%)	54 (7.4%)
**Patient Sex**						
Female (N = 1,055)	724 (68.6%)	331 (31.4%)	266 (80.9%)	82 (24.8%)	27 (8.2%)	25 (7.6%)
Male (N = 1281)	878 (68.5%)	403 (31.5%)	299 (74.4%)	103 (25.6%)	29 (7.2%)	29 (7.2%)
**Age Group**						
<1 y/o (N = 1,006)	726 (72.2%)	280 (27.8%)	180 (64.5%)	107 (38.2%)	23 (8.2%)	36 (12.9%)
1-4 y/o (N = 995)	648 (65.1%)	347 (34.9%)	295 (85.3%)	60 (17.3%)	17 (5.0%)	14 (4.0%)
5-14 y/o (N = 335)	228 (68.1%)	107 (31.9%)	90 (84.9%)	18 (16.8%)	16 (14.9%)	4 (3.7%)
**Median age in years (IQR)**	1.2 (0.3, 3.1)	1.3 (0.6, 3.2)	1.6 (0.8, 3.6)	0.8 (0.3, 1.9)	1.4 (0.4, 5.2)	0.8 (0.2, 1.1)
**Any risk factor**						
No (N = 1,726)	1,244 (72.1%)	482 (27.9%)	378 (78.8%)	111 (23.0%)	25 (5.2%)	31 (6.4%)
Yes (N = 300)	151 (50.3%)	149 (49.7%)	109 (73.6%)	44 (29.5%)	20 (13.4%)	19 (12.7%)
Unknown (N = 310)	207 (66.8%)	103 (33.2%)	78 (75.7%)	30 (29.1%)	11 (10.6%)	4 (3.9%)
**Asthma**						
No (N = 1,862)	1,271 (68.3%)	591 (31.7%)	458 (77.5%)	163 (27.6%)	40 (6.8%)	36 (6.1%)
Yes (N = 109)	47 (43.1%)	62 (56.9%)	41 (66.1%)	22 (35.5%)	6 (9.7%)	4 (6.5%)
**Chronic respiratory disease**						
No (N = 1,870)	1,275 (68.2%)	595 (31.8%)	454 (76.3%)	168 (28.2%)	37 (6.2%)	32 (5.4%)
Yes (N = 104)	45 (43.3%)	59 (56.7%)	48 (81.4%)	17 (28.8%)	7 (11.9%)	9 (15.3%)
**Neuromuscular disease**						
No (N = 1,929)	1,295 (67.1%)	634 (32.9%)	488 (77.0%)	181 (28.5%)	40 (6.3%)	36 (5.7%)
Yes (N = 42)	28 (66.7%)	14 (33.3%)	7 (50.0%)	4 (28.6%)	4 (28.6%)	5 (35.7%)
**Immunodeficiency**						
No (N = 1,941)	1,297 (66.8%)	644 (33.2%)	493 (76.6%)	184 (28.6%)	44 (6.8%)	40 (6.2%)
Yes (N = 29)	24 (82.8%)	5 (17.2%)	3 (60.0%)	1 (20.0%)	0 (0.0%)	1 (20.0%)
**Chronic cardiac disease**						
No (N = 1,938)	1,303 (67.2%)	635 (32.8%)	485 (76.4%)	181 (9.4%)	43 (6.8%)	38 (6.0%)
Yes (N = 29)	16 (55.2%)	13 (44.8%)	11 (84.6%)	4 (30.8%)	2 (15.4%)	2 (15.4%)
**Diabetes**						
No (N = 1,958)	1,311 (67.0%)	647 (33.0%)	495 (76.5%)	185 (28.6%)	44 (6.8%)	40 (6.2%)
Yes (N = 8)	8 (100.0%)	0 (0.0%)	0 (0.0%)	0 (0.0%)	0 (0.0%)	0 (0.0%)
**Renal insufficiency**						
No (N = 1,955)	1,309 (67.0%)	646 (33.0%)	494 (76.5%)	185 (0.5%)	44 (6.8%)	40 (6.2%)
Yes (N = 8)	6 (75.0%)	2 (25.0%)	2 (100.0%)	0 (0.0%)	0 (0.0%)	0 (0.0%)
**Hepatic insufficiency**						
No (N = 1,961)	1,314 (67.0%)	647 (33.0%)	495 (76.5%)	185 (28.6%)	44 (6.8%)	40 (6.2%)
Yes (N = 1)	1 (100.0%)	0 (0.0%)	0 (0.0%)	0 (0.0%)	0 (0.0%)	0 (0.0%)
**Obesity**						
No (N = 1,958)	1,313 (67.1%)	645 (32.9%)	493 (76.4%)	184 (28.5%)	43 (6.7%)	40 (6.2%)
Yes (N = 4)	2 (50.0%)	2 (50.0%)	2 (100.0%)	1 (50.0%)	1 (50.0%)	0 (0.0%)

* Cases might have several complications. ** Row wise percentage calculation.
SARI: severe acute respiratory infection, y/o: years old, ICU: intensive care unit admission, ARDS: Acute respiratory distress syndrome.

1.6 SUPPLEMENTARY TABLE S6: OCCURRENCE OF COMPLICATIONS AMONG CHILDREN <15 Y/O HOSPITALIZED WITH SARI IN BELGIUM DURING INFLUENZA SEASONS 2011-2012 TO 2019-2020, PER INFLUENZA TYPE, SUBTYPE AND LINEAGE.

	Total cases (N = 2,336)	Influenza negative (N = 1,568)	Influenza positive (N = 768)	Type A amongst positive (N = 565)	Type B amongst positive (N = 203)	H1N1 amongst type A (N = 341)	H3N2 amongst type A (N = 203)	Unidentified type A (N = 21)	Victoria amongst type B (N = 91)	Yamagata amongst type B (N = 107)	Unidentified lineage B (N = 5)
**Cases with any type of complication***	734 (31.4%)	554 (35.3%)	180 (23.4%)	137 (24.2%)	43 (21.2%)	90 (26.4%)	38 (18.7%)	9 (42.9%)	16 (17.6%)	26 (24.3%)	1 (20.0%)
**Type of complication among cases with complications**											
Pneumonia	565 (77.0%)	406 (73.3%)	159 (88.3%)	121 (88.3%)	38 (88.4%)	80 (88.9%)	33 (86.8%)	8 (88.9%)	15 (93.8%)	22 (84.6%)	1 (100.0%)
Respiratory Assistance	185 (25.2%)	152 (27.4%)	33 (18.3%)	26 (19.0%)	7 (16.3%)	17 (18.9%)	9 (23.7%)	0 (0.0%)	3 (18.8%)	4 (15.4%)	0 (0.0%)
ICU	56 (7.6%)	43 (7.8%)	13 (7.2%)	10 (7.3%)	3 (7.0%)	4 (4.4%)	4 (10.5%)	2 (22.2%)	2 (12.5%)	1 (3.8%)	0 (0.0%)
ARDS	54 (7.4%)	41 (7.4%)	13 (7.2%)	11 (8.0%)	2 (4.7%)	9 (10.0%)	2 (5.3%)	0 (0.0%)	1 (6.3%)	1 (3.8%)	0 (0.0%)

* Cases might have several complications.

SARI: severe acute respiratory infection, y/o: years old, ICU: intensive care unit admission, ARDS: Acute respiratory distress syndrome.

1.7 SUPPLEMENTARY TABLE S7: UNIVARIATE AND MULTIVARIABLE ANALYSIS OF THE RISK FOR COMPLICATIONS AMONG INFLUENZA POSITIVE CHILDREN <15 Y/O HOSPITALIZED WITH SARI IN BELGIUM DURING INFLUENZA SEASONS 2011-2012 TO 2019-2020.

**Total** (N = 673)	**No complication**	**Complication**	**Univariate RR (95% CI)**	**P-value**	**Multivariable OR (95% CI)***	**P-value**
**Influenza type**						
A	137	38	1			
B	371	126	0.86 (0.62, 1.18)	ns	0.92 (0.66, 1.25)	ns
**Risk Factor**						
No	463	117	1			
Yes	46	47	2.51 (1.91, 3.22)	<0.0001	2.85 (2.17,3.64)	<0.0001
**Sex**						
Female	253	92	1			
Male	256	73	0.84 (0.64, 1.10)	ns	0.90 (0.69,1.15)	ns
**Patient age in years**			0.97 (0.92, 1.01)	ns	0.94 (0.89, 0.98)	<0.01

* Model adjusted for influenza type, risk factor, patient sex and age.
SARI: severe acute respiratory infection, y/o: years old, RR = risk ratio, CI = confidence interval, ns = not significant.

1.8 Supplementary Table S8: Identified respiratory viruses in children hospitalized with SARI in Belgium during influenza seasons 2015-2016 to 2019-2020.

		Influenza infection status								Age group			Complications		Risk factors		
	**Total (N = 1,701)**	**Negative (N = 1,130)**	**Positive (N = 571)**	**Type B (N = 154)**	**Type A (N = 417)**	**H1N1 (N = 276)**	**H3N2 (N = 133)**	**Victoria (N = 90)**	**Yamagata (N = 61)**	**<1 y/o (N = 745)**	**1-4 y/o (N = 721)**	**5-14 y/o (N = 235)**	**No (N = 1210)**	**Yes (N = 491)**	**No (N = 1272)**	**Yes (N = 212)**	**Unknown (N = 217)**
**Any other respiratory viruses than influenza**	981 (57.7%)	838 (74.2%)	143 (25.0%)	34 (22.1%)	109 (26.1%)	69 (25.0%)	36 (27.1%)	20 (22.2%)	14 (23.0%)	517 (69.4%)	404 (56.0%)	60 (25.5%)	667 (55.1%)	314 (64.0%)	754 (59.3%)	121 (57.1 %)	106 (48.8%)
**Picorna-viruses**	384 (39.1%)	330 (39.4%)	54 (37.8%)	13 (38.2%)	41 (37.6%)	28 (40.6%)	11 (30.6%)	8 (40.0%)	5 (35.7%)	198 (38.3%)	167 (41.3%)	19 (31.7%)	273 (22.6%)	111 (22.6%)	291 (38.6%)	50 (41.3 %)	43 (40.6%)
**Adenovirus**	252 (25.7%)	207 (24.7%)	45 (31.5%)	10 (29.4%)	35 (32.1%)	23 (33.3%)	10 (27.8%)	5 (25.0%)	5 (35.7%)	114 (22.1%)	125 (30.9%)	13 (21.7%)	173 (14.3%)	79 (16.1%)	202 (26.8%)	26 (21.5 %)	24 (22.6%)
**hMPV**	234 (23.9%)	212 (25.3%)	22 (15.4%)	7 (20.6%)	15 (13.8%)	10 (14.5%)	5 (13.9%)	7 (35.0%)	0 (0.0%)	115 (22.2%)	102 (25.2%)	17 (28.3%)	144 (11.9%)	90 (18.3%)	179 (23.7%)	28 (23.1 %)	27 (25.5%)
**RSV A or B**	162 (16.5%)	146 (17.4%)	16 (11.2%)	3 (8.8%)	13 (11.9%)	6 (8.7%)	7 (19.4%)	3 (15.0%)	0 (0.0%)	118 (22.8%)	41 (10.1%)	3 (5.0%)	104 (8.6%)	58 (11.8%)	128 (17.0%)	14 (11.6 %)	20 (18.9%)
**Bocavirus**	138 (14.1%)	118 (14.1%)	20 (14.0%)	5 (14.7%)	15 (13.8%)	9 (13.0%)	5 (13.9%)	1 (5.0%)	4 (28.6%)	70 (13.5%)	66 (16.3%)	2 (3.3%)	86 (7.1%)	52 (10.6%)	103 (13.7%)	17 (14.0 %)	18 (17.0%)
**Corona-viruses OC43, NL63 or 229E**	98 (10.0%)	81 (9.7%)	17 (11.9%)	3 (8.8%)	14 (12.8%)	9 (13.0%)	5 (13.9%)	2 (10.0%)	1 (7.1%)	59 (11.4%)	34 (8.4%)	5 (8.3%)	74 (6.1%)	24 (4.9%)	73 (9.7%)	13 (10.7 %)	12 (11.3%)
**Para-influenza viruses**	82 (8.4%)	73 (8.7%)	9 (6.3%)	4 (11.8%)	5 (4.6%)	2 (2.9%)	3 (8.3%)	2 (10.0%)	2 (14.3%)	42 (8.1%)	32 (7.9%)	8 (13.3%)	54 (4.5%)	28 (5.7%)	60 (8.0%)	13 (10.7 %)	9 (8.5%)

SARI: severe acute respiratory infection, y/o: years old.

1.9 SUPPLEMENTARY TABLE S9: UNIVARIATE AND MULTIVARIABLE ANALYSIS OF THE RISK FOR COMPLICATIONS AMONG INFLUENZA-POSITIVE AND -NEGATIVE CHILDREN <15 Y/O HOSPITALIZED WITH SARI IN BELGIUM DURING INFLUENZA SEASONS 2011-2012 TO 2019-2020.

**Total** (N = 2,026)	**No complication**	**Complication**	**Univariate RR (95% CI)**	**P-value**	**Multivariable OR (95% CI)***	**P-value**
**Influenza infection status**						
Positive	509	164	1		1	
Negative	886	467	1.42 (1.22, 1.65)	<0.001	1.38 (1.18, 1.61)	<0.0001
**Risk Factor**						
No	1244	482	1		1	
Yes	151	149	1.78 (1.55, 2.04)	<0.001	1.71 (1.47, 1.98)	<0.0001
**Sex**						
Female	644	286	1		1	
Male	751	345	1.02 (0.90, 1.16)	ns	1.02 (0.90, 1.16)	ns
**Patient age in years**			0.99 (0.98, 1.0)	ns	1.00 (0.98, 1.02)	ns

* multivariable analysis adjusting for sex, influenza infection status negative or positive, risk factors, age in years. Children with unknown status of risk factor were excluded from this analysis.

SARI: severe acute respiratory infection, y/o: years old, RR = risk ratio, CI = confidence interval, ns = not significant.

2 Supplementary Methods

2.1 Study design and data collection

A retrospective analysis of the surveillance data on SARI in hospitalized children in Belgium under the age of 15 years (<15 y/o) was conducted. Routine data collection took place during the influenza epidemic periods 2011/2012 to 2019/2020, through the national Severe Acute Respiratory Infection (SARI) sentinel hospital surveillance network consisting of six hospitals in Belgium as described previously.[1] Information on complications, such as occurrence of pneumonia or acute respiratory distress syndrome (ARDS), need for respiratory assistance, transfer to an intensive care unit (ICU), or extracorporeal membrane oxygenation (ECMO), as well as on underlying risk factors for severe disease, such as asthma, chronic respiratory or cardiac disease, neuromuscular disease, immunodeficiency, diabetes, renal or hepatic insufficiency, and obesity, was collected via questionnaire.

2.2 Laboratory testing

Respiratory specimen were collected in an exhaustive manner at the time of data collection, and were sent to the National Influenza Center (NIC) at Sciensano for virological analysis as previously described.[2] Since 2015 a range of other respiratory viruses are included in the protocol.

2.3 Study population

All respiratory specimen received by the NIC through the national Severe Acute Respiratory Infection (SARI) sentinel hospital surveillance network during influenza season 2011-2012 to 2019-2020 were eligible. Specimen were excluded from the study population if information on any of the patient's dates of symptom start, admission, sampling or outcome were missing. Then, only specimen from patients who fulfilled the study case definition were included: acute respiratory illness with onset within the last ten days, with measured or reported fever of ≥38°C, with cough and/or dyspnoea, and with hospitalization for at least overnight. This case definition was adapted from the 2013 WHO case definition for SARI. Patients older than 15 years, or with missing age, or sex were then excluded. Finally, only individuals with complete information on complications were included in this specific part of the analysis.

2.4 Data analysis tools and R packages

All data analysis was performed in R (version 4.0.3), in R Studio. Differences in proportions between groups were compared by two proportion z-test using the prop.test() function with Yates continuity, Pearson's Chi-squared Test using the chisq.test() function or Fishers' exact test using the fisher.test() function in the stats package (version 4.0.2). Chi-squared test for trend was performed using the prop_trend_test() function in the rstatix package (version 0.7.0). The median and interquartile range (IQR) of years of age and days of hospitalization per influenza type and subtype or lineage, were calculated and compared using the function pairwise.wilcox.test() with Benjamini Hochberg corrections for multiple testing in the stats package. Univariate analysis was performed using the epi.2by2 function in the epiR package (version 1.0-15) to calculate the risk ratio (RR) and 95% confidence interval (CI) for developing complications among cases were calculated.

3 Supplementary References

1 Subissi L, Bossuyt N, Reynders M, *et al.* Spotlight influenza: Extending influenza surveillance to detect non-influenza respiratory viruses of public health relevance: Analysis of surveillance data, Belgium, 2015 to 2019. *Eurosurveillance* 2021; **26**: 1–10.

2 Fischer N, Dauby N, Bossuyt N, *et al.* Monitoring of human coronaviruses in Belgian primary care and hospitals , 2015 – 20 : a surveillance study. *The Lancet Microbe* 2021; **5247**: 1–10.
